# Fecal microbiota transplantation for treatment of refractory or recurrent *Clostridioides difficile* infection in Taiwan: a cost-effectiveness analysis

**DOI:** 10.3389/fmed.2023.1229148

**Published:** 2023-10-02

**Authors:** Kai-Yen Lan, Puo-Hsien Le, Cheng-Tang Chiu, Chien-Chang Chen, Yuan-Ming Yeh, Hao-Tsai Cheng, Chia-Jung Kuo, Chyi-Liang Chen, Yi-Ching Chen, Pai-Jui Yeh, Cheng-Hsun Chiu, Chee-Jen Chang

**Affiliations:** ^1^Department of Biomedical Sciences, Chang Gung University College of Medicine, Taoyuan, Taiwan; ^2^Department of Gastroenterology and Hepatology, Chang Gung Memorial Hospital, Linkou Branch, Taoyuan, Taiwan; ^3^Taiwan Association of the Study of Small Intestinal Disease, Taoyuan, Taiwan; ^4^Chang Gung Microbiota Therapy Center, Chang Gung Memorial Hospital, Linkou Branch, Taoyuan, Taiwan; ^5^Liver Research Center, Chang Gung Memorial Hospital, Linkou Branch, Taoyuan, Taiwan; ^6^Department of Pediatric Gastroenterology, Chang Gung Memorial Hospital, Linkou Branch, Taoyuan, Taiwan; ^7^Division of Gastroenterology and Hepatology, Department of Internal Medicine, New Taipei Municipal Tucheng Hospital, New Taipei City, Taiwan; ^8^Molecular Infectious Disease Research Center, Chang Gung Memorial Hospital, Linkou Branch, Taoyuan, Taiwan; ^9^Division of Pediatric Infectious Diseases, Department of Pediatrics, Chang Gung Memorial Hospital, Linkou Branch, Taoyuan, Taiwan; ^10^Graduate Institute of Clinical Medicine, Chang Gung University College of Medicine, Taoyuan, Taiwan; ^11^Department of Obstetrics and Gynecology, Chang Gung Memory Hospital, Linko Branch, Taoyuan, Taiwan; ^12^Research Service Center for Health Informatics, Chang Gung University, Taoyuan, Taiwan

**Keywords:** *Clostridioides difficile*, inflammatory bowel disease, cost-effectiveness analysis, economic evaluation, fecal microbiota transplantation, vancomycin, fidaxomicin

## Abstract

**Background:**

Compared to antibiotic treatment, fecal microbiota transplantation (FMT) is a more effective treatment for refractory or recurrent CDI (rCDI). Patients with inflammatory bowel disease (IBD) have a higher incidence of CDI and worse outcomes. There has been no study from Asia to evaluate the cost-effectiveness of FMT for overall rCDI patients and rCDI patients with IBD.

**Methods:**

We applied a Markov model with deterministic and probabilistic sensitivity analyses to evaluate the cost and effectiveness of different treatments for rCDI patients with a time horizon of 1 year from the payer's perspective. We compared the cost and clinical outcomes of FMT through colonoscopy to two antibiotics (vancomycin and fidaxomicin) using data from Chang Gung Memorial Hospital, Taoyuan, Taiwan.

**Results:**

Compared to vancomycin, FMT was cost-effective in overall rCDI patients as well as IBD patients with rCDI [USD 39356 (NT$1,101,971.98)/quality-adjusted life year (QALY) gained in overall patients; USD65490 (NT$1,833,719.14)/QALY gained in IBD patients]. Compared to fidaxomicin, FMT was only cost-effective in overall rCDI patients [USD20255 (NT$567,133.45)/QALY gained] but slightly increased QALY (0.0018 QALY gained) in IBD patients with rCDI.

**Conclusion:**

FMT is cost-effective, compared to vancomycin or fidaxomicin, for the treatment of rCDI in most scenarios from the payers' perspective in Taiwan.

## Introduction

*Clostridioides difficile* infection (CDI) is the most common healthcare-associated infection (1). It can cause severe diarrhea, toxic megacolon, and bowel perforation with a high mortality rate (2). The risk factors include antibiotic exposure, older age, longer hospital stay, previous CDI, and inflammatory bowel disease (IBD) (3). Although the incidence of IBD in Asia is lower than in Europe and North America, the incidence has been rapidly increasing within the past decade (4). In patients with IBD, the incidence rate of CDI was 2–8 times higher than in the general population due to mucosal defects and medications (5). CDI not only leads to acute flare-ups but also negatively impacts clinical outcomes, including therapeutic escalation, more hospitalizations, emergent department visits, surgeries, and even higher mortality in IBD (5). Therefore, the treatment of CDI is a very important issue in the management of IBD.

Antibiotic treatment is effective in treating CDI, but the recurrence rate is up to 25% within 30 days after completing treatment (6). Fidaxomicin can be used for the treatment of recurrent CDI (6, 7). Compared to antibiotic treatment, single fecal microbiota transplantation (FMT) provides a nearly 90% success rate with a much lower recurrence rate for refractory or recurrent CDI (rCDI) (7). Therefore, FMT is strongly recommended in treating rCDI according to updated guidelines (8–10). The U.S. Food and Drug Administration approved the first fecal microbiota product, Rebyota, in November 2022. Additionally, Vowst, the first orally administered fecal microbiota product, has also received approval. They are indicated for the prevention of recurrent CDI in adults, following antibacterial treatment for recurrent CDI. The incidence of FMT-related adverse events is low and most are self-limited. Because FMT is more expensive than antibiotic treatments, the cost-effectiveness analysis from the payer's perspective is important to develop healthcare policies.

Some studies from Western countries indicated that FMT is cost-effective, compared to antibiotic treatments (vancomycin, metronidazole, and fidaxomicin) in the general population with rCDI (10–16). Only one study showed its cost-effectiveness in IBD patients with rCDI in Hong Kong when compared to vancomycin (with or without bezlotoxumab) and fidaxomicin based on the therapeutic results of global studies (17). However, the common strains of *C. difficile* in Asia are different from the strains in Europe and North America (18). Hence, we need a pharmacoeconomic analysis based on Asian cases. In this study, we aimed to provide the first cost-effectiveness analysis of FMT in both the general population and IBD patients with rCDI, based on the local therapeutic results in Taiwan.

## Methods

### Model design

**Figure 1:**
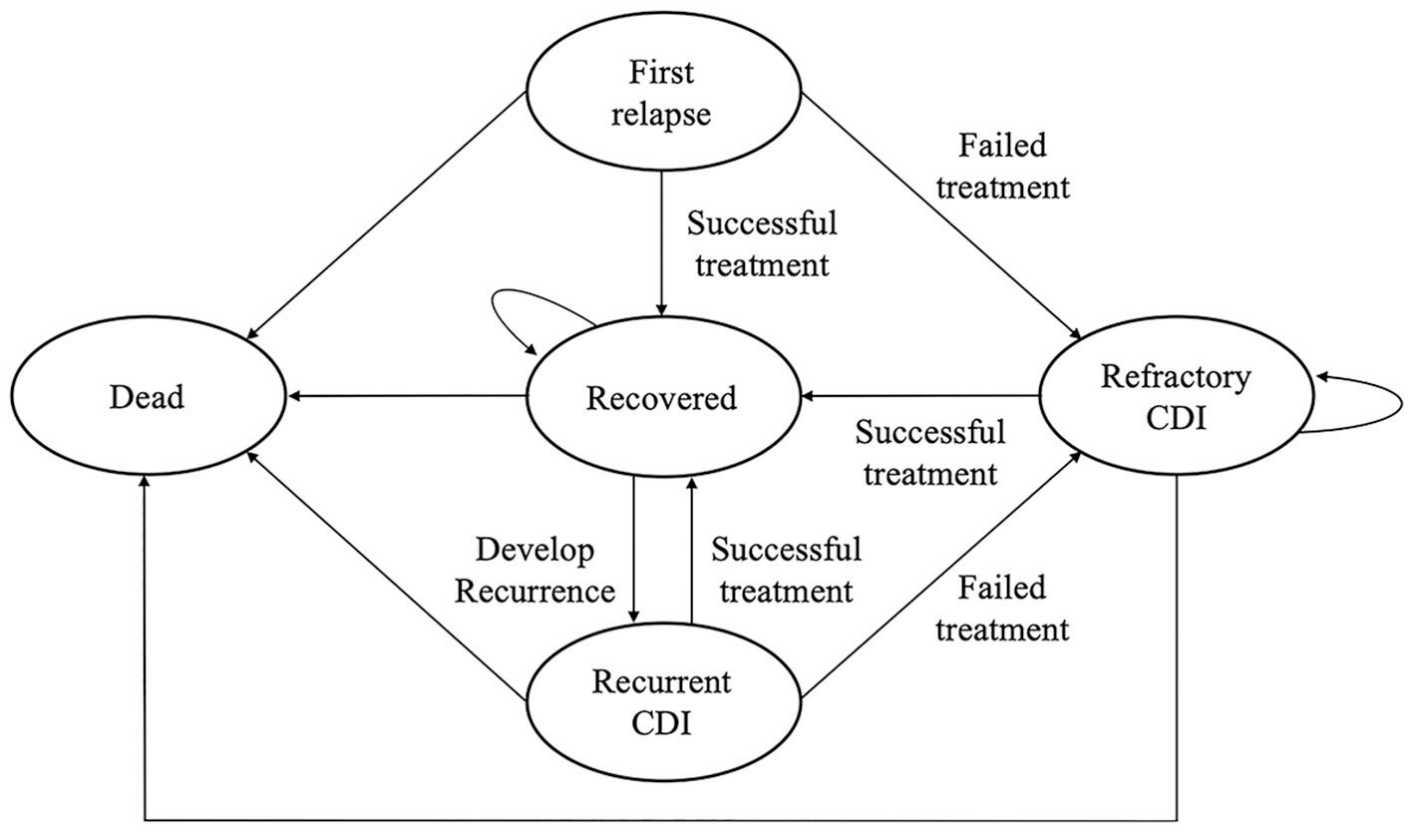
A Markov model is used to investigate the cost-effectiveness of FMT vs. antibiotics in the treatment of refractory or recurrent *Clostridioides difficile* infection in Taiwan.

We constructed a Markov model to evaluate the cost-effectiveness of FMT through colonoscopy in rCDI in a 1-year time horizon from the payer's perspective. A Markov model was used because of the high recurrence rate of CDI. We assumed the model consisted of five statuses, including first relapse, recovery, refractory CDI, recurrent CDI, and death (Figure 1) using TreeAge Pro 2020 (TreeAge Software, Williamstown, Massachusetts, USA). Fidaxomicin is administered in the event of recurrence following the initial vancomycin or after initial vancomycin failure (vancomycin arm). FMT is employed when patients experience a recurrence after the initial fidaxomicin or encounter initial fidaxomicin failure (fidaxomicin arm). In the case of FMT failure or the recurrence of CDI after the initial FMT cure (FMT arm), repeat FMT is considered.

FMT was compared to standard treatment with vancomycin (125 mg four times a day for 10 days) or fidaxomicin (200 mg twice a day for 10 days) in treating rCDI (8). In this study, the length of each cycle was 3 months. A half-cycle correction was applied to account for the fact that events in the model could happen at any point. Incremental cost-effectiveness ratio (ICER) and net monetary benefit (NMB) were used to conclude the results of the comparisons.

### Clinical inputs

The clinical outcomes of FMT were based on the preliminary outcome report of Chang Gung Microbiota Therapy Center (CGMTC), Chang Gung Memorial Hospital (19, 20). The diagnosis of CDI relies on detecting the presence of toxins A and B in the stool. A cure is achieved when symptoms are resolved and there are two consecutive negative test results for toxins A and B. The cure and recurrence rates of vancomycin and fidaxomicin in overall CDI patients and CDI patients with IBD were collected from the available evidence using a pragmatic review. The data were extracted to merge within different weightings in different population numbers and the weighted average was re-calculated as probabilities. All clinical parameters are shown in Table 1 (11, 21–29).

**Table 1:** Transition probabilities, costs, and utilities in this study.

**Category**	**Base-case**	**Lower**	**Upper**	**Distribution**	**Reference**
**Cure rate**					
Vancomycin	87.5%	81.6%	93.4%	Beta (α:105, β:15)	(21–23)
Vancomycin (IBD)	78.6%	57.1%	99%	Beta (α: 11, β: 3)	(24)
FMT	90.5%	84.2%	96.8%	Beta (α: 76, β: 8)	CGMTC
FMT (IBD)	87.5%	76%	99%	Beta (α: 28, β: 4)	CGMTC
Fidaxomicin	89.7%	83.3%	96.1%	Beta (α: 79, β: 9)	(21, 22)
Fidaxomicin (IBD)	81.3%	69.6%	93%	Beta (α: 35, β: 8)	(25, 26)
**Recurrence rate**					
Vancomycin	31.0%	24.8%	38.2%	Beta (α: 32, β: 72)	(21–23)
Vancomycin (IBD)	45.5%	16.1%	74.9%	Beta (α: 5, β: 6)	(24)
FMT	9.21%	2.71%	15.7%	Beta (α: 7, β: 69)	CGMTC
FMT (IBD)	27.0%	10.6%	43.4%	Beta (α: 5, β: 14)	CGMTC
Fidaxomicin	20.3%	11.4%	29.2%	Beta (α: 16, β: 63)	(21, 22)
Fidaxomicin (IBD)	22.8%	8.9%	36.7%	Beta (α: 8, β: 27)	(25, 26)
Crude mortality rate (3 m)	0.2%	fixed	fixed	fixed	Local data
Mortality from CDI	0.7%	0.2%	1.2%	Beta (α:12, β:1,724)	(12, 27)
**Utility**					
Improve	1	Fixed	Fixed	Fixed	-
Dead	0	Fixed	Fixed	Fixed	-
Recurrent CDI	0.82	0.72	0.84	Triangular (0.82, 0.72, 0.84)	(11, 28, 29)
Refractory CDI	0.71	0.5	0.72	Triangular (0.71,0.50,0.72)	(11, 28, 29)
**Cost**					
Vancomycin	25,160	18,870	31,450	Gamma (25, 160, SD: 3,145)	Local cost
FMT	67,000	50,250	83,750	Gamma (67,000, SD: 8,375)	Local cost
FMT (recurrence)	67,286	50,465	84,108	Gamma (67,286, SD: 8,411)	Local cost
Fidaxomicin	21,627	16,220	27,034	Gamma (21,627, SD: 2,703)	Local cost

CGMTC, Chang Gung Microbiota Therapy Center; CDI, *Clostridioides difficile* infection; FMT, fecal microbiota transplantation; IBD, inflammatory bowel disease; SD, standard deviation.

The costs of vancomycin, fidaxomicin, FMT via colonoscopy, and the costs of the ward, registration, diagnostic, medicine service, and nursing fees were included in this analysis.

All costs are expressed in New Taiwan Dollars (NT$).

### Cost inputs

The detailed information on the cost parameters was according to the National Health Insurance Administration, Directorate-General of Budget, Accounting and Statistics, and CGMTC, Chang Gung Memorial Hospital, Linkou. All costs are shown in the New Taiwan Dollar (NT$).

### Utility inputs

Utility is converted from the scores of perceived health status. The utilities of improvement, refractory, and recurrence were 1.0, 0.71, and 0.82, respectively (28–30).

### Outputs and sensitivity analyses

The results were presented as ICER and NMB, which were the ratio that expresses the results and the rearrangement expression of a cost-effectiveness analysis.

ICERs were calculated as follows:


$$\begin{array}{l} ICER=\frac{\left(Cost_{FMT}-Cost_{Antibiotics}\right)}{\left(Effectiveness_{FMT}-Effectiveness_{Antibiotics}\right)} \end{array}$$

NMBs were calculated as follows:


$$\begin{array}{l} NMB=\text{λ}\times \left(Effectiveness_{FMT}-Effectiveness_{Antibiotics}\right)\\ \;-\left(Cost_{FMT}-Cost_{Antibiotics}\right) \end{array}$$

λ were set as three-times (3x) GDP per capita (NT$2,929,920).

The gross domestic product (GDP) per capita of Taiwan in 2021 was NT$976640 according to the Directorate-General of Budget, Accounting and Statistics, Executive Yuan of R.O.C (Taiwan). The willingness-to-pay (WTPs, λ) was set as three-times (3x) GDP per capita (NT$2,929,920). Within this range, FMT was defined as cost-effective.

Sensitivity analyses were done to determine the uncertainty and the robustness of the model. Both deterministic sensitivity analysis (DSA) and probabilistic sensitivity analysis (PSA) were performed in this study. All parameters including clinical transition probabilities, utilities, costs, corresponding distributions, and references are listed in Table 1. We used a 95% confidence interval to define the lower and upper bounds of the parameters of probability. As for costs, the ranges were defined as between 25% below and above the base-case values. In DSA, we chose one-way sensitivity analysis to estimate the changes in tornado diagrams. In PSA, we selected beta distribution (probabilities), gamma distribution (costs), and triangular distribution (utilities). Approximately 5,000 Monte Carlo simulations were generated from all distributions of the interventions, and 1,000 Monte Carlo simulations were shown on the PSA scatter plots. Finally, the results of PSA were demonstrated as scatter plots and acceptability curves.

## Results

### Base-cases

A Markov model (Figure 1) was constructed to compare FMT with vancomycin and fidaxomicin from a payer's perspective with a time horizon of 1 year. The results of the base-case analysis are presented in Table 2. FMT was the most effective treatment in all scenarios, and it had a QALY of 0.9170 to 0.9363. Vancomycin was the cheapest intervention, which costs NT$29498.08–36,825.00 in different scenarios.

**Table 2:** Base-case results of strategies for refractory or recurrent *Clostridioides difficile* infection.

**Category**	**Cost**	**Incr cost**	**QALY**	**Incr QALY**	**ICER**	**NMB**
**FMT vs. vancomycin**						
FMT (Overall)	54,713.28	25,215.20	0.9363	0.0229	1,101,971.98	41,879.968
Vancomycin (Overall)	29,498.08	-	0.9134	-	-	-
FMT (IBD)	80,643.52	43,818.52	0.9170	0.0239	1,833,719.14	26206.568
Vancomycin (IBD)	36,825.00	-	0.8931	-	-	-
**FMT vs. fidaxomicin**						
FMT (Overall)	54,713.28	6,512.67	0.9363	0.0115	567,133.45	27,181.41
Fidaxomicin (Overall)	48,200.61	-	0.9248	-	-	
FMT (IBD)	80,643.52	22,716.11	0.9170	0.0018	12,360,976.83	−17,442.254
Fidaxomicin (IBD)	57,927.41	-	0.9152	-	-	-

FMT, fecal microbiota transplantation; IBD, inflammatory bowel disease; Incr, incremental; QALY, quality-adjusted life year; ICER, incremental cost-effectiveness ratio; NMB, Net monetary benefit.

All costs are expressed in New Taiwan Dollars (NT$).

FMT was cost-effective, compared to vancomycin in overall rCDI patients and rCDI patients with IBD (NT$1,101,971.98/QALY gained in overall rCDI patients; NT$1,833,719.14/QALY gained in rCDI patients with IBD). When we compared FMT to fidaxomicin, it was also cost-effective in overall rCDI patients (NT$567,133.45/QALY gained), but not in rCDI patients with IBD (NT$12,360,976.83/QALY gained). In brief, FMT was cost-effective in all scenarios except when comparing FMT and fidaxomicin in rCDI patients with IBD, and it also had slight QALY gained (0.0018 QALY) in this group of patients.

### Deterministic sensitivity analysis

**Figure 2:**
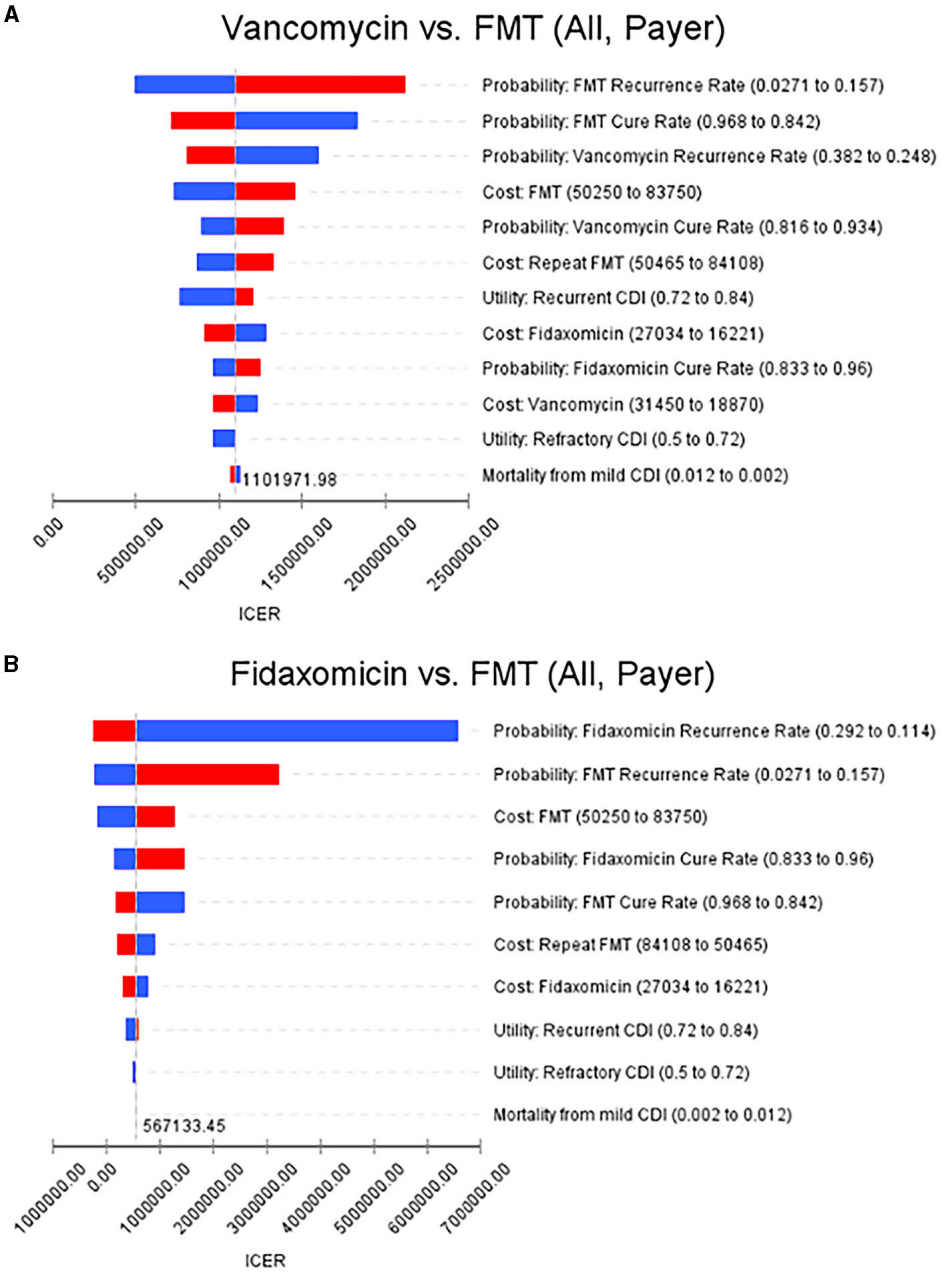
Tornado diagrams show the results of the deterministic sensitivity analyses. **(A)** FMT vs. vancomycin in overall rCDI patients from the payer's perspective. **(B)** FMT vs. fidaxomicin in overall rCDI patients from the payer's perspective (increases are shown in red and decreases in blue, when the parameter values were changed). FMT, fecal microbiota transplantation; rCDI, refractory or recurrent *Clostridioides difficile* infection.

The recurrence rate of FMT had the greatest influence on ICERs, and FMT was still cost-effective in overall rCDI patients after adjusting to the upper bound of the recurrence rate. Moreover, ICERs were not affected by the mortality rate in overall rCDI patients in this model. As for rCDI patients with IBD, the recurrence rate of vancomycin had the greatest influence on ICERs when we compared FMT to vancomycin. In comparing FMT to fidaxomicin, the cure and recurrence rates had an extreme influence on ICERs which exceeded NT$20,000,000/QALY gained. The details are illustrated in Figures 2A, B.

### Probabilistic sensitivity analysis

**Figure 3:**
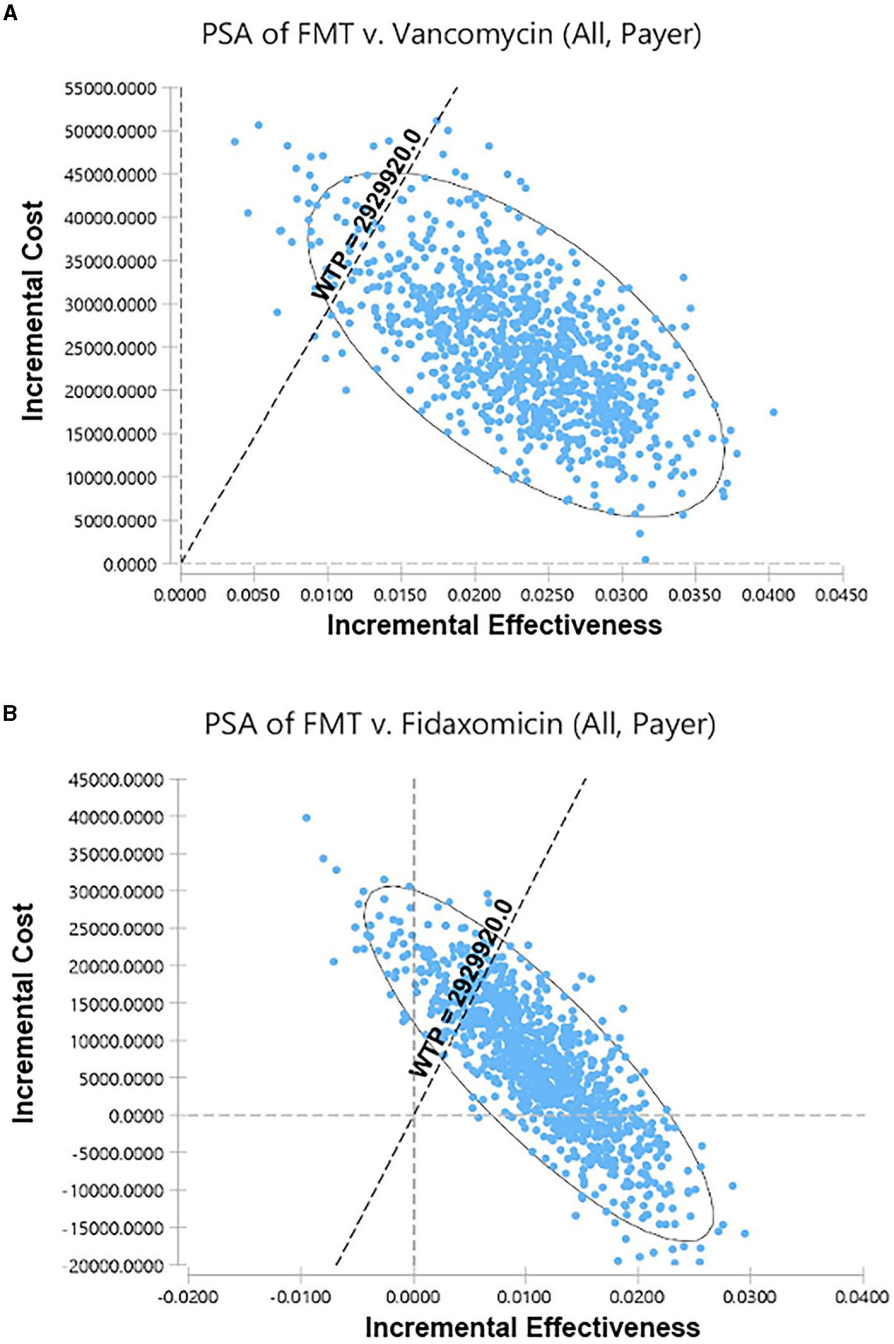
Scatter plots for the probabilistic sensitivity analyses. **(A)** FMT vs. vancomycin in overall rCDI patients from the payer's perspective. **(B)** FMT vs. fidaxomicin in overall rCDI patients from the payer's perspective. FMT, fecal microbiota transplantation; rCDI, refractory or recurrent *Clostridioides difficile* infection; WTP, willingness-to-pay.

**Figure 4:**
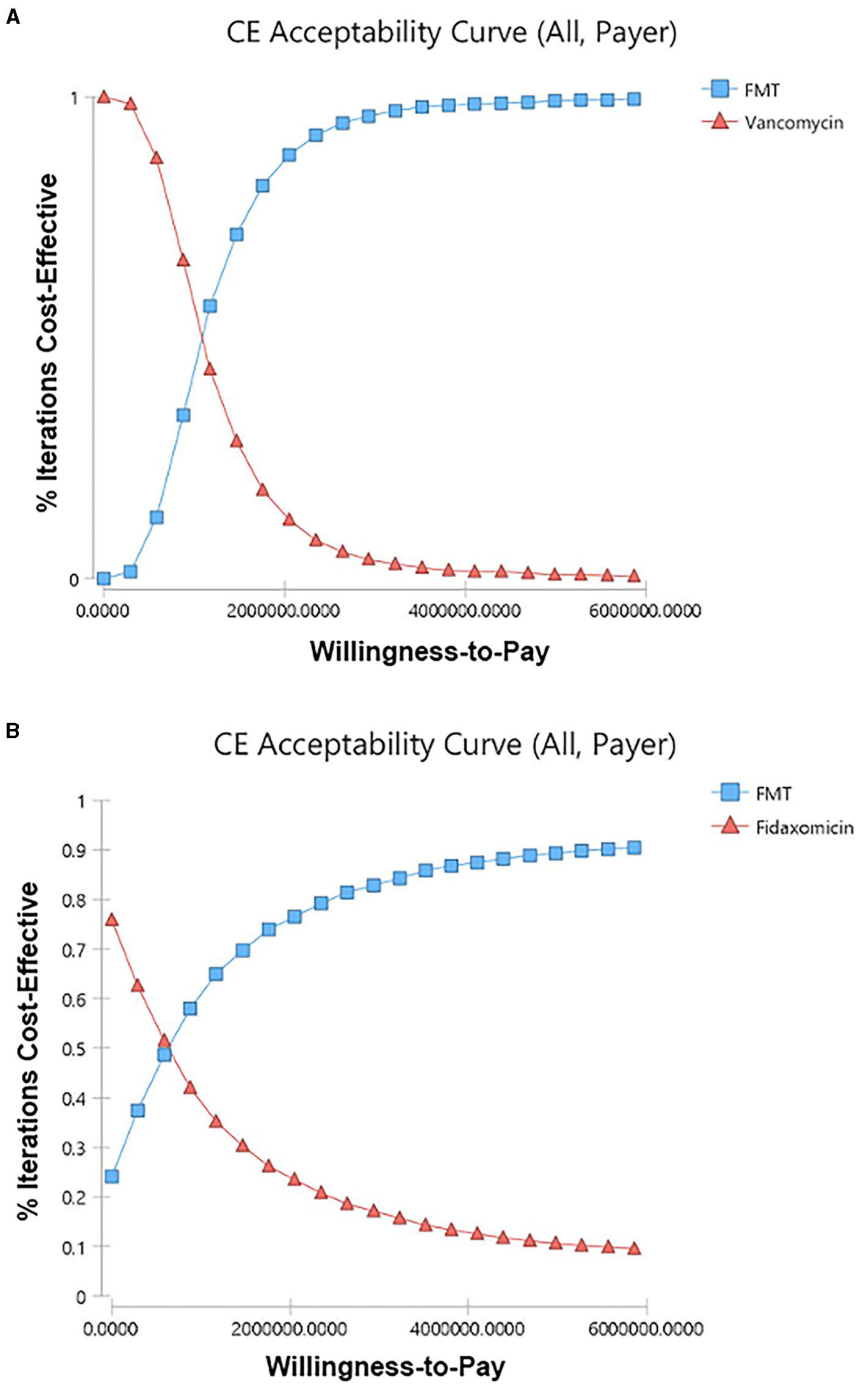
Cost-effectiveness acceptability curves. **(A)** FMT vs. vancomycin in overall rCDI patients from the payer's perspective. **(B)** FMT vs. fidaxomicin in overall rCDI patients from the payer's perspective. CE, cost-effectiveness. FMT, fecal microbiota transplantation; rCDI, refractory or recurrent *Clostridioides difficile* infection.

Among 5,000 simulations, 95.9% of them were grouped under a threshold which indicated that FMT is cost-effective compared to vancomycin in overall rCDI patients (Figure 3A). Compared to fidaxomicin, FMT was also cost-effective in overall rCDI patients with 83.28% simulations grouped under threshold (Figure 3B). However, only 67.54% and 36.28% of simulations favored FMT when we compared it to vancomycin and fidaxomicin in rCDI patients with IBD. Acceptability curves of the scenarios in overall CDI patients are shown in Figures 4A, B.

## Discussion

FMT is superior to antibiotics for the treatment of rCDI, but it is more expensive (7). The cost-effective analysis from the payer's perspective is crucial for health authorities to create a reimbursement policy for FMT in rCDI. Despite the limited number of cases, variations in antibiotic and FMT costs, and the heterogeneous study designs of published FMT-related economic evaluation studies across different countries, the latest systematic review published in 2020 concludes that FMT appears to be a cost-effective treatment for rCDI (31). However, only one study from Hong Kong based on publicly available data revealed superior cost-effectiveness of FMT in IBD patients with rCDI, relative to antibiotic treatment (17). Some earlier studies from Western countries used a simulating, hypothetical cohort of patients for analysis (14). We provided the first pharmacoeconomic study that compared FMT to antibiotics (vancomycin and fidaxomicin) in overall rCDI patients and rCDI patients with IBD based on real-world, local data in Asia. Compared to antibiotic treatments, we found FMT was cost-effective in overall CDI patients from the payer's perspective in Taiwan. The higher cure rate and lower recurrence rate of FMT can explain the result.

In rCDI patients with IBD, FMT was still cost-effective when compared to vancomycin but not fidaxomicin treatment in this study. However, it has been shown cost-effective in a study from Hong Kong, compared to fidaxomicin based on the therapeutic results from Western countries (32). Four reasons can explain the difference. First, there was only slight QALY gained in rCDI patients with IBD who received FMT in this study. Second, the cure rate (FMT, 87.5%; fidaxomicin, 81.3%) and recurrence rate (FMT, 27%; fidaxomicin, 22.8%) of FMT were not much higher than fidaxomicin treatment (Table 1). Third, the cost of fidaxomicin (NT$57927) was significantly lower than FMT (NT$80642) in Taiwan. Finally, FMT led to a lower recurrence rate than antibiotic treatment, and a time horizon of 1 year might be not long enough to highlight the advantage. Furthermore, we also performed the same analysis from the societal perspective, and the results were similar.

The limitations of the study included mixed refractory and recurrent *C. difficile* infections, mixed Crohn's disease and ulcerative colitis, and a short time horizon of 1 year. Furthermore, our parameter estimates relied on one single center protocol with a relatively small size in a preliminary report (19). However, there are no uncertainties regarding cost and service, as we set up a fecal bank to provide quality microbiota transplants for the service (19). We continue providing FMT material for rCDI cases even during the COVID-19 pandemic with the same protocol and at listed prices. As of February 2023, more than 100 cases have been treated by FMT in Chang Gung Memorial Hospital with a similar successful rate (Chiu CH, unpublished data). Nevertheless, further pharmacoeconomic studies are needed to understand the cost-effectiveness of all available strategies for different rCDI subgroups and in different countries.

## Conclusion

Compared to antibiotic treatments, FMT is cost-effective in the treatment of rCDI in most scenarios from the payer's perspective in Taiwan. From this perspective, our findings support the growing body of clinical evidence from Asian countries that FMT can be used to treat rCDI. The study also provides timely and useful information for health authorities to develop health and insurance policies for FMT.

## Data availability statement

The raw data supporting the conclusions of this article will be made available by the authors, without undue reservation.

## Ethics statement

The studies involving humans were approved by the Institutional Review Board (IRB) of the Chang Gung Medical Foundation (approval document no. 202201148B0. Cost Effectiveness Analysis of Fecal Microbiota Transplantation in Recurrent or Refractory *Clostridioides difficile* Infection in Taiwan) for the period from 10 August 2022 to 9 August 2023. The studies were conducted in accordance with the local legislation and institutional requirements. Written informed consent for participation was not required from the participants or the participants' legal guardians/next of kin in accordance with the national legislation and institutional requirements.

## Author contributions

K-YL: methodology, validation, formal analysis, investigation, data curation, writing—original draft, and visualization. P-HL: methodology, software, validation, formal analysis, investigation, writing—original draft, and visualization. C-TC and C-CC: methodology, software, validation, and formal analysis. Y-MY: software, validation, formal analysis, investigation, and data curation. H-TC: validation, formal analysis, investigation, and data curation. C-JK: formal analysis, resources, and investigation. C-LC: data curation, validation, and investigation. Y-CC: formal analysis, resources, and software. P-JY: data curation, resources, and investigation. C-HC: conceptualization, investigation, and writing—review and editing. C-JC: conceptualization, methodology, and writing—review and editing. All authors contributed to the article and approved the submitted version.
